# IL27Rα Deficiency Alters Endothelial Cell Function and Subverts Tumor Angiogenesis in Mammary Carcinoma

**DOI:** 10.3389/fonc.2019.01022

**Published:** 2019-10-04

**Authors:** Annika F. Fink, Giorgia Ciliberti, Rüdiger Popp, Evelyn Sirait-Fischer, Ann-Christin Frank, Ingrid Fleming, Divya Sekar, Andreas Weigert, Bernhard Brüne

**Affiliations:** ^1^Faculty of Medicine, Institute of Biochemistry I, Goethe-University Frankfurt, Frankfurt, Germany; ^2^Faculty of Medicine, Institute for Vascular Signalling, Goethe-University Frankfurt, Frankfurt, Germany

**Keywords:** IL-27 cytokine, endothelial cell, mammary cancer, cytokine, angiogenesis

## Abstract

IL-27 regulates inflammatory diseases by exerting a pleiotropic impact on immune cells. In cancer, IL-27 restricts tumor growth by acting on tumor cells directly, while its role in the tumor microenvironment is still controversially discussed. To explore IL-27 signaling in the tumor stroma, we used a mammary carcinoma syngraft approach in IL27Rα-deficient mice. Tumor growth in animals lacking IL27Rα was markedly reduced. We noticed a decrease in immune cell infiltrates, enhanced tumor cell death, and fibroblast accumulation. However, most striking changes pertain the tumor vasculature. Tumors in IL27Rα-deficient mice were unable to form functional vessels. Blocking IL-27-STAT1 signaling in endothelial cells *in vitro* provoked an overshooting migration/sprouting of endothelial cells. Apparently, the lack of the IL-27 receptor caused endothelial cell hyper-activation via STAT1 that limited vessel maturation. Our data reveal a so far unappreciated role of IL-27 in endothelial cells with importance in pathological vessel formation.

## Introduction

Interleukin 27 (IL-27) is a heterodimeric cytokine of the IL-12 family, composed of IL-27p28 and Epstein–Barr virus (EBV)-induced gene 3 (EBI3). It is mainly expressed and secreted by antigen presenting cells. IL-27 signals via a receptor complex, consisting of IL27Rα and the signal-transducing glycoprotein 130 (gp130) (1, 2). Gp130 is found in a number of receptor complexes, including the IL-6 receptor. Therefore, specificity of IL-27 signaling depends on IL27Rα. IL27Rα is expressed on many immune and stromal cells, whereas it is nearly absent on B cells and neutrophils. Once IL-27 binds to its receptor complex, mainly janus kinase (JAK) and downstream signal transducer and activator of transcription (STAT) are activated (3, 4).

IL-27 regulates inflammation by acting, among others, on T cells. Its pleiotropic functions are shaped by a given inflammatory environment. IL-27 can enhance Th1 immunity by suppressing Th2/Th17 cell development (5, 6), but also acts immune-suppressive, e.g., by upregulating inhibitory immune checkpoint receptors, such as PD-L1 and CTLA4 (7, 8). Consequently, IL-27 affects a number of diseases. IL27Rα-deficient mice treated with a high dose of dextran sulfate sodium (DSS) elevated Th17 cell activity, translating into aggravated colitis. In contrast, IL-27 application in an acute colitis model attenuated disease outcome (9, 10). Moreover, IL-27 delayed the onset of experimental autoimmune encephalomyelitis (EAE), which was attributed to enhanced IL-10 expression and downstream suppression of IL-17 production (11). Indeed, the absence of IL27Rα aggravated EAE outcome, with increased Th17 cell numbers (12).

Also, the impact of IL-27 on tumor development revealed divergent effects. IL-27 overexpressing C26 colon carcinoma cells induced interferon γ (IFNγ) expression in splenic cells, promoting antitumor activity by augmenting CD8^+^ T cells (13). In addition to potential immune-stimulatory effects, IL-27 directly inhibited proliferation and tumorigenicity of human prostate cancer cells (hPCa) *in vitro* (14), as well as *in vivo* in a xenograft mouse models with hPCa cells or human multiple myeloma cells (14, 15). Immune cell independent effects were also suggested when IL-27 inhibited the growth of subcutaneously implanted B16-F10 melanomas, in wildtype (WT) as well as IFNγ-deficient or NOD-SCID mice. In this setting, IL-27 restricted B16-F10 pulmonary metastasis by inducing the production of the antiangiogenic chemokines CXCL10 or CXCL9 from HUVECs (16). However, a tumor-promoting role of IL-27 has also been proposed. IL-27 induced immune-suppressive molecules in stromal cells, including immune checkpoint molecules and CD39 (17, 18). To further explore the role of IL-27 in tumor stromal cells, we used a mammary carcinoma cell syngraft approach in IL27Rα-deficient mice. While our data confirm a tumor-promoting role of IL-27 in the tumor stroma, we uncovered an unexpectedly strong impact of IL-27 signaling on the tumor vasculature. The absence of IL-27 signaling severely limits the formation of functional blood vessels and thus, tumor angiogenesis.

## Materials and Methods

### Reagents

Epigallocatechin gallat (EGCG), Stattic and lipopolysaccharide (LPS) were purchased from Sigma-Aldrich (St. Louis, USA). IFNγ was from BioVision (Milpitas, USA). IL-4 was from Peprotech (Hamburg, Germany). IL-27 was obtained from Biolegend (Koblenz, Germany), IL-27 neutralizing antibody was from Invitrogen (Carlsbad, USA), and the IgG2a istotype control was from BioXCell (West Lebanon, USA). Macrophage colony-stimulating factor (M-CSF) and granulocyte-macrophage colony-stimulating factor (GM-CSF) were from ImmunoTools (Friesoythe, Germany). All reagents were dissolved according to the manufacturer's instructions.

### Cell Culture

The murine endothelial cell line bEnd5 was obtained from the HPA Culture Collections via Sigma-Aldrich in August 2018. Experiments with these cells were completed within 3 months and the cells were therefore not authenticated again. bEnd5 cells were cultured in DMEM (Thermo Fisher Scientific, Waltham, USA) containing 1% sodium pyruvate (Sigma-Aldrich) and 1% non-essential amino acids (Sigma-Aldrich). Fibroblast 3T3 cells were cultured in DMEM/F-12 medium (Thermo Fisher Scientific). Murine breast cancer cells (PyMT) were cultured in DMEM containing 1% sodium pyruvate, 1% non-essential amino acids, and 10 mmol/L HEPES (Sigma-Aldrich). Media was supplemented with 10% FCS (Capricorn Scientific, Epsdorfergrund, Germany), 100 U/ml penicillin, and 100 μg/ml streptomycin (PAA laboratories, Cölbe, Germany).

### Animal Experiments

Murine breast cancer cells derived from a mouse expressing the *polyoma virus middle T oncoprotein* (PyMT) under the mouse mammary tumor virus promoter were transplanted into four mammary glands of IL27Rα wildtype (WT) and knockout (KO) mice. Tumor growth was monitored for up to 31 days until tumors reached a diameter of 1.5 cm in WT animals. Tumor volume was calculated as follows: volume = 0.5 × (length × width^2^). After 21 or 31 days, mice were euthanized followed by cardiac perfusion with 0.9% NaCl solution and tumors were harvested. Animal experiments followed the guidelines of the Hessian animal care and use committee (approval No. FU/1106).

### Flow Cytometry

Single suspensions of tumors were generated using the mouse tumor dissociation kit and the gentleMACS dissociator (Miltenyi Biotec, Bergisch Gladbach, Germany). Single cell suspensions were stained with fluorochrome-coupled antibodies and analyzed by flow cytometry using an LSRII Fortessa cell analyzer (BD Biosciences, Heidelberg, Germany). Data were analyzed using FlowJo software VX (Treestar, Ashland, USA). Antibodies were titrated to determine optimal concentrations. For single-color compensation CompBeads (BD Bioscience) were used to create multi-color compensation matrices. Cells were blocked with 2% Fc Receptor Binding Inhibitor (Miltenyi) in PBS for 10 min on ice. Afterwards, cells were stained for either analyzing the immune cell composition, or for characterizing endothelial cells. To discriminate immune cell subsets in tumors the following Abs were used: anti-CD3-PE-CF594 (BD); anti-CD4-BV711 (BD); anti-CD8-BV650 (Biolegend); anti-CD11b-BV605 (Biolegend); anti-CD11c-BV711 (BD); anti-CD19-APC-H7 (BD); anti-CD25-PE-Cy7 (BD); anti-CD44-AlexaFluor700 (BD); anti-CD45-VioBlue (Miltenyi Biotec); anti-CD326-BV711 (BD); anti-GITR-FITC (Biolegend); anti-F4/80-PE-Cy7 (Biolegend); anti-Ly-6C-PerCP-Cy5.5 (BD); anti-Ly-6G-APC-Cy7 (BD); anti-NK1.1-BV510 (BD). To define endothelial cell (EC) populations the following Abs were used: anti-CD45-AlexaFluor700 (BD); anti-CD326-BV711 (BD); anti-CD31-PE-Cy7 (eBioscience); anti-CD204-PE (Miltenyi); anti-LYVE-1-PE (R&D system); anti-CD90.2-PE (Miltenyi); anti-CD146-AlexaFluor488 (BD); anti-ICAM1(CD54)-BV421 (BD); anti-CD62P(P-selectin)-BV510 (BD); anti-CD62E(E-selectin)-BV650 (BD); anti-CD109(VCAM1)-PerCP-Cy5.5 (Biolegend); anti-CD141(Thrombomodulin)-APC (Novus, Wiesbaden, Germany).

### Histology and Immunohistochemistry

Tumors and lungs were zinc fixed and paraffin-embedded. Tumor sections were stained using the Opal staining system and analyzed with InForm software using the phenotyping tool according to the manufacturer's instructions (PerkinElmer, Rodgau, Germany). Tumor sections were stained with the following antibodies: cleaved caspase (Cell Signaling, Cambridge, U.K.); Ki67 (abcam, Cambridge, U.K.); hypoxia-inducible factor 1-alpha (HIF1α) (Novus); panCytokeratin (abcam); CD31 (BD); alpha smooth muscle actin (αSMA) (Sigma-Aldrich); spectral DAPI (PerkinElmer); neural/glial antigen 2 (NG2) (R&D systems, Minneapolis, USA). For metastases at least nine independent sections of each lung were stained with Mayer's hemalum (Merck, Darmstadt, Germany) and analyzed. Secondary antibody controls for each antibody species were routinely included (Supplementary Figure 1).

### BSA-FITC Vessel Permeability Assay

FITC labeled BSA (50 mg/kg) (Sigma-Aldrich) was injected i.p. 90 min prior to sacrificing mice. FITC-dependent fluorescence was visualized together with CD31 as indicated above. The FITC-positive area was analyzed using ImageJ.

### Isolation and Generation of Bone Marrow Derived-Macrophages (BMDM)

For the generation of BMDMs, femur and tibia of WT and KO mice were extracted. BM cells were plated in RPMI 1640 medium containing 20 ng/ml GM-CSF and 20 ng/ml M-CSF. Cells were incubated for 7 days. Afterwards cells were exposed to 100 ng/ml LPS, 10 ng/ml IFNγ, 20 ng/ml IL-4 or directly co-cultured with PyMT cells.

### RNA Isolation and Quantitative Real-Time PCR

RNA from tumor samples were isolated using the PeqGold RNAPure™ protocol (Peqlab Biotechnologie, Erlangen, Germany) and transcribed into cDNA using Fermentas Reverse Transcriptase Kit (Thermo Fisher Scientific). Quantitative Real-Time PCR was performed using the SYBR green and the MyIQ real-time PCR system (Bio-Rad, Munich, Germany). The following primers were used from Biomers (Ulm, Germany): mouse ubiquitin-40S ribosomal protein S27a (*Rps27a*) forward 5′-GACCCTTACGGGGAAAACCAT-3′, reverse 5′-AGACAAAGTCCGGCCATCTTC-3′; mouse *Ki67* forward 5′-ACCGTGGAGTAGTTTATCTGGG-3′, reverse 5′-TGTTTCCAGTCCGCTTACTTCT-3′; mouse proliferating cell nuclear antigen (*Pcna*) forward 5′-TTTGAGGCACGCCTGATCC-3′, reverse 5′-GGAGACGTGAGACGAGTCCAT-3′; mouse collagen type 1 alpha 1 chain (*Col1a1*) forward 5′-GCTCCTCTTAGGGGCCACT-3′, reverse 5′-CCACGTCTCACCATTGGGG-3′; mouse collagen type 3 alpha 1 chain (*Col3a1*) forward 5′-AAGGCTGCAAGATGGATGCT-3′, reverse 5′-GTGCTTACGTGGGACAGTCA-3′; mouse alpha smooth muscle actin (*Acta2*) forward 5′-CCCAGACATCAGGGAGTAATGG-3′, reverse 5′-TCTATCGGATACTTCAGCGTCA-3′; mouse fibronectin 1 (*Fn1*) forward 5′-TCAGAAGAGTGAGCCCCTGA-3′, reverse 5′-AAGATTGGGGTGTGGAAGGG-3′; mouse mannose receptor C-type 1 (*Mrc1*) forward 5′-GGAGTGATGGAACCCCAGTG-3′, reverse 5′-CTGTCCGCCCAGTATCCATC-3′; mouse arginase 1 (*Arg1*) forward 5′-GTGAAGAACCCACGGTCTGT-3′, reverse 5′-CTGGTTGTCAGGGGAGTGTT-3′; mouse transglutaminase 2 (*Tgm2*) forward 5′-AGAGTGTCGTCTCCTGCTCT-3′, reverse 5′-GTAGGGATCCAGGGTCAGGT-3′; mouse inducible nitric oxide synthases (*Nos2*) forward 5′-ACCCTAAGAGTCACAAAATGG-3′, reverse 5′-TTGATCCTCACATACTGTGGACG-3′; mouse *IL27R*α forward 5′-GGACCAGGAAACCATTGGAGT-3′, reverse 5′-GTTGAGCTTGTCCAGGCTGTC-3′; mouse *IL-27p28* forward 5′-CAGGGCTATGTCCACAGCTT-3′, reverse 5′-CGAAGTGTGGTAGCGAGGAA-3′.

Primers for mouse vascular endothelial growth factor A (*Vegf*), *Il10*, and tumor necrosis factor α (*Tnf*-α) were from QuantiTect (Qiagen, Hilden, Germany).

### siRNA Transfection

To analyze the impact of the IL27Rα chain on endothelial cells, bEnd5 cells were transfected either with IL27Rα siRNA or control siRNA (Dharmacon, Lafayette, USA) using HiPerfect (Qiagen) according to the manufacturer's instructions.

### Generation of Tumor Supernatants

Tumors were crushed with mortar and pestle in liquid nitrogen. Two times 2 × PBS of the tumor weight was added to the crushed tumors and the suspension was incubated for 3 h at 4°C under rotation. After centrifugation, the supernatant and the cell pellet were used for further experiments.

### Cytokine Quantification

To analyze cytokines in bEnd5 cell culture supernatants and tumor extracellular fluid (19), the LEGENDplex Mouse cytokine panel 2 was used (Biolegend) according to the manufacturer's instructions. Samples were acquired by flow cytometry and analyzed using FlowJo VX.

### Immunoblotting

Tumor cell pellets were sonified in HIF-lysis buffer (6.65 M Urea, 10% glycerol, 1% SDS, 10 mM Tris; pH 7.4), 100 ng protein per sample was loaded on SDS polyacrylamid gels together with SDS loading buffer (0.5 M Tris, pH 6.8; 2% SDS, 20% glycerol, 0.002% bromphenol blue, 5 mM DTT). Proteins were blotted on a nitrocellulose membrane, incubated with β-actin (Sigma-Aldrich), phospho-STAT (pSTAT1) (Cell Signaling), total STAT1 (tSTAT1) (Cell Signaling), pSTAT3 (Cell Signaling), tSTAT3 (Cell Signaling), and visualized by IRDye 680- and IRDye 800-coupled secondary Abs using the Li-Cor Odyssey imaging system (LICOR Biosciences, Bad Homburg, Germany).

### Enzyme-Linked Immunosorbent Assay

An ELISA for VEGF (R&D systems) was used to quantify VEGF in tumor supernatants. Tumor supernatants were generated as descripted above and diluted 1:50. ELISA was performed according to the manufacturer's instructions.

### Aortic Ring Assay

The aortic ring sprouting assay was performed as previously (20). Briefly, aortas were harvested from 8 to 10 weeks old mice and washed with DMEM/F14 medium (Gibco, Carlsbad, USA) supplemented with 100 U/mL penicillin, and 100 μg/mL streptomycin. The dissected aortas were subsequently cleaned, sectioned in 12–16 rings of 1 mm length, and embedded in collagen type 1 (Corning, New York, USA). After polymerization of the collagen gel, microvascular endothelial cell growth medium (PeloBiotech, Planegg, Germany) supplemented with 100 U/mL penicillin, 100 μg/mL streptomycin, and 2% murine serum (BD) was added into the well. Tube-like structures were allowed to develop over 7 days. Thereafter, samples were fixed in 4% PFA and endothelial cells were visualized using antibodies against CD31 (Dianova, Hamburg, Germany) and VE-Cadherin (R&D), while NG2 (Merck, Darmstadt, USA) staining was employed to detect pericytes. The total volume of vascular and perivascular sprouting in each explant was calculated trough the IMARIS-BITPLANE 9.3 software. Additionally, total sprout length was measured with ImageJ.

### Wound Healing Assay

To study endothelial cell migration, a wound healing assay using bEnd5 cells was performed. Cells were grown until they reached confluence. Afterwards the wound was created with a 10 μl pipette tip. To analyze the impact of IL-27 signaling, an IL-27 neutralizing antibody (1 ng/ml), an IgG2a istotype control (1 ng/ml), Stattic (50 ng/ml), Epigallocatechin gallat (EGCG) (10 ng/ml), siControl, or IL27Rα siRNA were used. Images were taken 16 and 24 h after wound generation and analyzed using the wound healing tool in ImageJ.

### Proliferation Assay

The IncuCyte® S3 live-cell analysis system (Sartorius, Göttingen, Germany) was used to study proliferation of bEnd5 endothelial cells. Images were taken every 4 h and the doubling time of cells was calculated as follows: doubling time = (duration + log2)/(log(final concentration) – log(initial concentration)).

### Statistics

Data are presented as means ± SEM. Statistical comparisons between two groups were performed using the Mann Whitney test, or paired/unpaired two-tailed Student's *t*-test as indicated. Data were pre-analyzed to determine normal distribution and equal variance with D'Agostino–Pearson omnibus normality test. Statistical analysis was performed with GraphPad Prism v8. Differences were considered significant at *p* < 0.05. No statistical test was used to predetermine sample size, and all samples were included in the analysis. Details on statistical tests used in individual experiments are found in the figure legends.

## Results

### Stromal IL-27 Signaling Promotes Mammary Tumor Growth and Reduces Immune Cell Infiltrates

**Figure 1:**
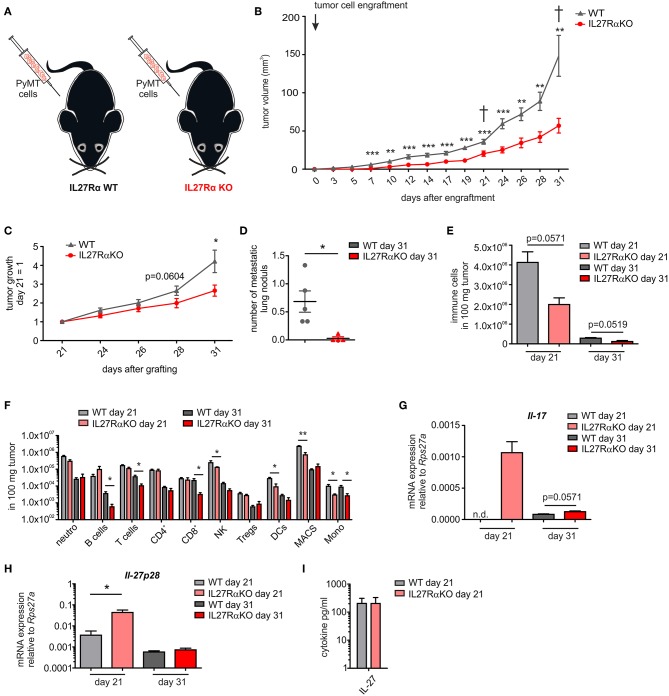
Tumor growth, metastasis, and immune cell composition are reduced in tumors of IL27Rα knockout KO mice. **(A)** PyMT breast cancer cells were transplanted into four mammary glands of IL27Rα wildtype (WT) and knockout (KO) mice, respectively. **(B)** Tumor onset and progression were time-dependently observed. **(C)** Tumor growth slope from day 21 to 31 was analyzed (WT *n* = 13, KO *n* = 17). **(D)** Lungs were harvested after 31 days and analyzed by immunohistochemistry (Mayer's hemalum staining) for metastasis occurrence. Nine section from independent regions of one lung lobe per animal were analyzed. Quantification shows the mean of these regions for each animal (WT *n* = 5, KO *n* = 4). **(E–G)** Immune cell composition of tumor single cell suspensions was analyzed using multicolor FACS analysis. **(E)** CD45^+^ immune cells are shown and **(F)** major immune cell subsets are displayed (WT day 21 *n* = 4, KO day 21 *n* = 4, WT day 31 *n* = 8, KO day 31 *n* = 8). **(G)** Quantitative real time PCR from whole tumor RNA for *Il-17* is given relative to *Rps27a* (*n* = 4). **(H)** Quantitative real time PCR from whole tumor RNA for *IL-27p28* is given relative to *Rps27a* (WT day 21 *n* = 4, KO day 21 *n* = 4, WT day 31 *n* = 6, KO day 31 *n* = 6). **(I)** IL-27 cytokine production within early stage tumor was analyzed using Legendplex (WT day 21 *n* = 4, KO day 21 *n* = 4). Data are means ± SEM, *p*-values were calculated using one-sample *t*-test; *p < 0.05, **p < 0.01, ***p < 0.001; n.d., not detected.

To analyze IL-27 signaling during breast cancer development, murine breast cancer cells derived from a polyoma middle T oncogene-driven primary tumor were transplanted into mammary glands of IL27Rα WT or KO mice (Figure 1A). Tumor growth was monitored up to 31 days (Figure 1B). Tumors transplanted into WT mice started to appear within the first week following transplantation, whereas the growth of tumors transplanted into mammary glands of IL27Rα KO mice was delayed. Moreover, tumor progression in IL27Rα KO mice was strongly reduced from day 21 onwards (Figure 1C). To explore mechanisms, mice were sacrificed at day 21 to analyze early stage tumors or at day 31, when first tumors in WT mice reached a pre-defined ethical end-point (tumor diameter of 1.5 cm). Analyzing earlier time points was not feasible due to low amounts of available tumor material. Correlating with reduced tumor growth, we also observed a lower number of pulmonary metastasis in IL27Rα KO mice with 31 days old tumors (Figure 1D).

The impact of IL-27 in tumors was so far attributed to direct suppression of tumor cells, or an altered immune cell infiltrate. Since tumor cells did not differ in their IL27Rα expression in both groups, we initially focused on immune cells. IL-27 augments the generation of cytotoxic T lymphocytes (CTL), blocks proliferation of CTL, activates natural killer (NK) cells and limits Th17 generation, but also promotes Treg expansion and/or activation (7, 8). To analyze changes in immune cell composition, tumor single cell suspensions were analyzed using multicolor FACS staining (Supplementary Figure 2). We observed a clear reduction of the overall immune cell infiltrate in tumors growing in IL27Rα KO compared to WT mice. This was apparent at early stage, as well as late stage tumors (Figure 1E) and affected all major immune cell subsets, with the notion that predominantly myeloid cells were affected at early and lymphocytic cells were affected at late stage (Figure 1F). Overall, a decrease in immune cell abundance in tumors was observed during tumor development, which is due to a decline in the acute response toward a transplanted tumor and an increase in tumor cells due to rapid proliferation. Importantly, we did not observe an increase of CTL or Tregs in tumors of IL27Rα KO mice. Quantitative PCR analysis revealed a minor increase in IL-17 mRNA in late stage tumors of IL27Rα KO mice, while a major increase was observed in early stage tumors of IL27Rα KO mice (Figure 1G), confirming an impact of IL-27 on Th17 generation. For control reasons, we evaluated the presence of IL-27 in tumors. IL-27 was expressed in tumors and there was an increase in the amount of IL-27 *p28* mRNA in early stage tumors growing in IL27Rα KO mice, which was, however, not observed at protein level (Figures 1H,I). There was no change in the expression of IL-27 *p28* mRNA in late stage tumors between WT and KO animals. The general decrease of IL-27 *p28* mRNA expression in late stage tumors can be explained by a reduced number of infiltrating immune cells, which are the main producers of IL-27. In conclusion, the overall reduction in immune cell infiltrates made it unlikely that specific lymphocyte subsets account for the altered tumor growth in IL27Rα KO mice.

### IL-27 and the IL-27 Receptor Do Not Directly Affect Macrophage Polarization

Tumor-associated macrophages (TAM) were the major immune cell population in tumors (Figure 1F). They decreased in early stage tumors of KO mice, following the decrease in their progenitors, i.e., monocytes (21). In late stage tumors of KO mice, they remained unaltered although monocyte numbers were still lower, indicating an uncoupling from recruitment into the tumors. This may be due to local proliferation (21). TAM may either support or restrict tumor growth based on their polarization state. Inflammatory M1-like macrophages show anti-tumor potential, whereas anti-inflammatory M2-like macrophages promote tumor development (22, 23). We explored polarization of macrophages by analyzing the expression of different macrophage markers at mRNA level. Within the bulk tumor mRNA, we detected a trend toward higher expression of M2-like macrophage markers in tumors of IL27Rα KO mice at early and late tumor stages (Supplementary Figure 3A). This pattern fits to a potential increase in proliferation, since macrophage proliferation was triggered by M2 stimuli (24). Expression of the classical M1 marker *Nos2* was significantly decreased in tumors of IL27Rα KO mice, which may suggest a reduced anti-tumor potential of TAM in tumors of IL27Rα KO mice. To study whether this was linked to IL-27-signaling in macrophages, we generated BMDM from WT and IL27Rα KO mice and induced classical activation with LPS/IFNγ, alternative activation with IL-4, or directly co-cultured them with PyMT cells to induce tumor-like conditions. All stimuli were applied with or without the addition of IL-27. Afterwards quantitative PCR analysis for alternatively activated macrophage/M2 markers (*Tgm2, Arg1, Mrc1*), the classically activated macrophage/M1 marker (*Nos2*), as well as the cytokines *Il10* and *Tnf*-α was performed (25, 26) (Supplementary Figures 3B–G). Stimulation of BMDM with LPS/IFNγ increased *Nos2, Tnf*-α, and *Il-10* expression, which was unaltered in KO BMDM or upon IL-27 addition. Stimulation with IL-4 enhanced expression of *Tgm2, Arg1*, and *Mrc1*, which was again independent of IL-27. Coculturing BMDM with PyMT cells upregulated *Arg1* and strongly suppressed *Tnf*-α expression, again with no impact of IL-27. Thus, IL27Rα-deficiency may restrict M1-like polarization in tumors, although tumors growing in IL27Rα KO mice were smaller. Therefore, these alterations were likely secondary and not due to IL-27 signaling directly in macrophages. In conclusion, we excluded macrophages as major players in reducing tumor growth in IL27Rα KO mice.

### Cancer-Associated Fibroblasts in Late Stage Tumors of IL27Rα KO Mice

Since immune cells did not explain reduced tumor growth in IL27Rα KO mice, we focused on other stromal cells. Tumor sections were stained for the tumor fibroblast marker αSMA. An unexpectedly large difference of αSMA-expressing cells between late stage, but not early stage tumors, was detected. In late stage tumors of KO mice significantly more αSMA positive fibroblasts were observed compared to WT mice (Supplementary Figures 4A,B). To analyze a potentially direct impact of IL-27 on fibroblasts, 3T3 murine fibroblasts were stimulated with transforming growth factor β (TGFβ) to induce a cancer-associated fibroblast phenotype (27), with or without the addition of IL-27, and several fibroblast activation and proliferation markers were analyzed (Supplementary Figure 4C). The mRNA expression of *Col1a1, Acta2*, as well as *Fn1* was upregulated after TGFβ treatment. Stimulation with IL-27 did not alter expression of these genes, although *Col3a1* was slightly decreased upon IL-27 stimulation. To analyze the effect of IL-27 toward fibroblast proliferation we analyzed the proliferation markers Ki67 and *Pcna* (Supplementary Figure 4C), but did not observe changes in proliferation when stimulating with TGFβ and/or IL-27. Apparently IL-27 signaling did not increase fibroblast numbers or activation *in vitro*.

### Increased Hypoxia in Tumor of IL27Rα KO Mice

**Figure 2:**
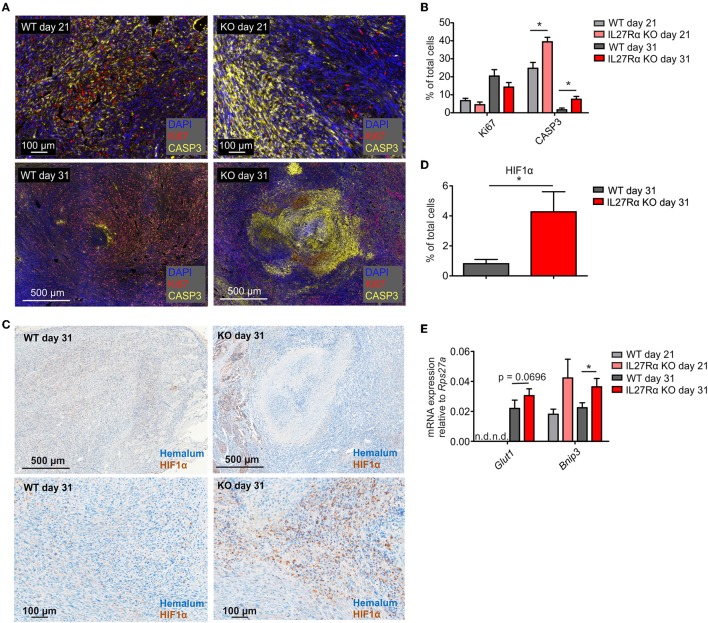
Increased hypoxia and tumor cell death in tumors of IL27Rα KO mice. PyMT breast cancer cells were transplanted into mammary glands of IL27Rα wildtype (WT) and knockout (KO) mice. Tumors were harvested after 21 or 31 days. **(A)** Immunohistochemistry for proliferation (Ki67) and apoptosis (active CASP3) in tumor sections of WT and KO mice. **(B)** Quantification of data shown in A using inForm Software (WT day 21 *n* = 4, KO day 21 *n* = 4, WT day 31 *n* = 8, KO day 31 *n* = 8). **(C)** Immunohistochemistry of HIF1α in tumor sections of WT and KO mice. **(D)** Quantification of data shown in C using inForm Software (WT *n* = 8, KO *n* = 8). **(E)** Expression of the Hif1α target genes *Glut1* and *Bnip3* was analyzed by quantitative real time PCR using whole tumor mRNA (WT day 31 *n* = 4, KO day 21 *n* = 4, WT day 31 *n* = 10, KO day 31 *n* = 11). Data are means ± SEM; *p*-values were calculated using one-sample *t*-test; *p < 0.05; n.d., not detected.

To understand reduced tumor growth in IL27Rα KO mice, we next analyzed proliferation and apoptosis of tumor cells. Tumor sections of late stage tumors were stained for proliferating (Ki67) and apoptotic tumor cells (cleaved CASP3). There was a tendency toward decreased proliferation of tumors growing in IL27Rα KO mice, and a major increase in apoptotic tumor cells in IL27Rα KO compared to WT mice, both in early and late-stage tumors (Figures 2A,B). Cancer cells can deregulate proliferation signals and become hyper-proliferative (28), which requires constant nutrient and oxygen supply. Oxygen availability in solid tumors is often limited. As a consequence, the α-subunits of hypoxia-inducible factors (HIF1 and 2) are stabilized. HIF transcription factors then induce a selected set of target genes to increase blood supply and restore oxygen levels (29). To explore this connection, late stage tumor sections were stained for HIF1α (30) (Figure 2C). We noticed significantly more hypoxic cells in tumors of IL27Rα KO mice, compared to WT mice (Figure 2D). This pattern was confirmed at the level of HIF1α target genes including Bcl-2/adenovirus E1B 19 kDa interacting protein 3 (*Bnip3*), which was significantly increased in late stage tumors in IL27Rα KO mice, while glucose transporter 1 (*Glut1*) increased in early and late stage tumors of IL27Rα KO mice (Figure 2E). As tumors of IL27Rα KO mice were more hypoxic, an impaired oxygen supply may account for reduced growth and increased tumor cell death.

### Altered Endothelial Cell Numbers and Vessel Structure in Tumors of IL27Rα KO Mice

**Figure 3:**
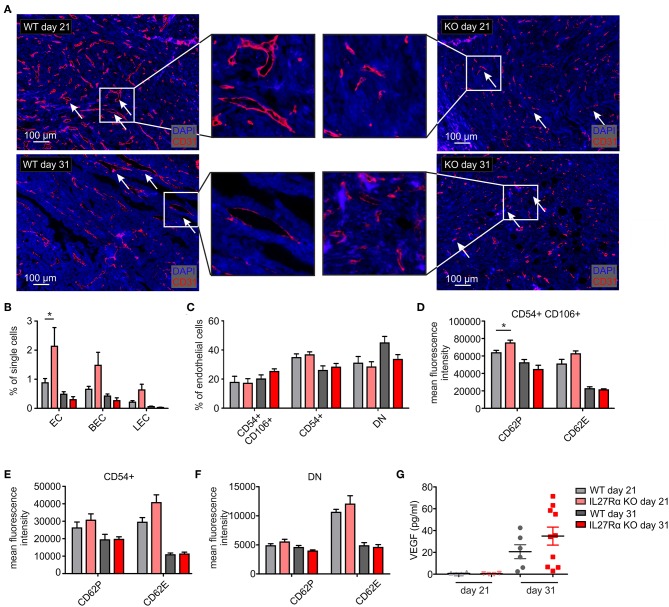
Altered endothelial cell architecture in tumors of IL27Rα KO mice. PyMT breast cancer cells were transplanted into four mammary glands of IL27Rα wildtype (WT) and knockout (KO) mice, respectively. Tumors were harvested after 21 or 31 days. **(A)** Representative immunohistochemical stainings of tumor sections for endothelial cells (CD31). **(B–F)** EC abundance **(B)** and activation status **(C–F)** were analyzed by flow cytometry. CD54 and CD106 expression **(C)** and the expression of cell surface markers CD62P, CD62E, and CD141 in CD54^+^ CD106^+^ **(D)**, CD54^+^ CD106^−^ **(E)** and double negative (DN) **(F)** EC populations were analyzed (WT day 21 *n* = 4, KO day 21 *n* = 4, WT day 31 *n* = 7, KO day 31 *n* = 7). **(G)** VEGF protein amount in whole tumor protein lysates analyzed by ELISA (WT day 21 *n* = 4, KO day 21 *n* = 4, WT day 31 *n* = 6, KO day 31 *n* = 10). Data are means ± SEM; *p*-values were calculated using one-sample *t*-test; *p < 0.05.

To better understand increased HIF1α expression and HIF-responses in tumors of IL27Rα KO mice, we analyzed the number and morphology of tumor blood vessels using immunofluorescence and multi-spectral FACS. Staining tumor sections of early and late stage tumors for the endothelial marker CD31 revealed marked differences in vessel architecture (Figure 3A). WT tumors contained well-structured vessels with a lumen, whereas vessels of IL27Rα KO mice were smaller, without luminal structures (arrows, Figure 3A). Often, single scattered CD31 positive cells were detected in tumors of KO mice (arrows, Figure 3A). Quantitative analysis by FACS showed that CD31^+^ ECs, both CD31^+^CD146^+^ blood endothelial cells (BEC) and CD31^+^CD90^+^LYVE1^+^ lymphatic endothelial cells (LEC), were increased in early stage, but not in late stage tumors of IL27Rα KO mice (Figure 3B). While EC infiltration was increased, immune cell infiltration was markedly decreased. Since immune cells interact with activated blood endothelial cells to infiltrate into tissues, EC activation in tumors was investigated using FACS (31). EC activation is characterized by cell-surface molecules, e.g., CD54 or CD106 (32). No major differences in EC subsets were detected, e.g., activated CD54^+^CD106^+^ cells, activated CD54^+^ or resting double negative (DN) cells (Figure 3C). However, activated CD54^+^CD106^+^ ECs of early IL27Rα KO tumors showed significantly more P-selectin (CD62P) and a tendency for increased E-selectin (CD62E) expression at the cell surface, which, however, was lost in late stage tumors (Figure 3D). Both molecules are essential for leukocyte recruitment. Their enhanced expression would be expected to increase immune cell interactions with ECs and cause immune cell recruitment, which did not correlate with our tumor phenotype. CD54 single positive cells and double negative resting cells showed no significant changes in P-selectin or E-selectin expression (Figures 3E,F). To understand the increase of EC in early stage IL27Rα KO tumors, we analyzed the expression of vascular endothelial growth factor-α, a HIF1α target gene and major pro-angiogenic growth factor (33). At protein level, we detected increased VEGF amounts in tumor supernatants of late stage tumors, but no changes between tumors growing in IL27Rα KO or WT mice (Figure 3G). These data suggested that altered VEGFA levels do not explain increased EC infiltration.

**Figure 4:**
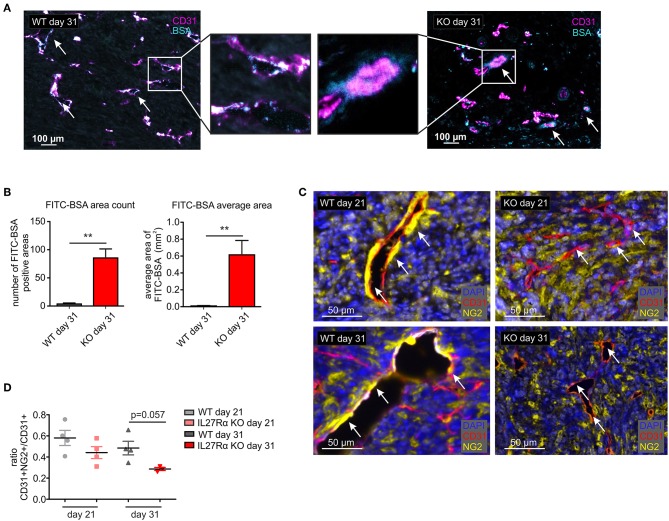
Vessel integrity and stability is decreased in tumors of IL27Rα KO mice. PyMT breast cancer cells were transplanted into mammary glands of IL27Rα wildtype (WT) and knockout (KO) mice. Tumors were harvested after 21 or 31 days. **(A)** Vessel integrity was determined at day 31 after tumor cell engraftment by injecting FITC labeled BSA (50 mg/kg) i.p. 90 min before sacrificing mice. FITC leakage was analyzed in combination with CD31 expression by immunohistochemistry. **(B)** BSA content within the tumors was analyzed using ImageJ, considering the count and the average size of FITC positive areas (WT *n* = 6, KO *n* = 7). **(C)** Immunohistochemistry of CD31 and NG2. **(D)** CD31 and NG2 expression was analyzed using ImageJ (WT day 21 *n* = 4, KO day 21 *n* = 4, WT day 31 *n* = 4, KO day 31 *n* = 3). Data are means ± SEM; *p*-values were calculated using one-sample *t*-test; **p < 0.01.

Since differences in vessel architecture could be observed, we next investigated vessel integrity within tumors. FITC-labeled BSA was injected i.p. 90 min before sacrificing tumor-bearing mice. Afterwards tumor sections were stained for CD31 by immunohistochemistry, combined with analysis of FITC fluorescence resulting from BSA leakage through the blood vessels into the tumor. In WT mice, BSA-FITC was mainly observed within tumor vessels, whereas in tumors of IL27Rα KO mice, FITC-BSA leaked into the tumor area surrounding vessels (arrows, Figure 4A). A significant increase in both, count and average size of BSA-FITC positive areas was apparent in tumors of IL27Rα KO compared to WT mice (Figure 4B). Vessel integrity is, among others, determined by coverage of the extra-luminal side of EC with pericytes (34). Given the leakiness of vessels in tumors of IL27Rα KO mice, we analyzed expression of the pericyte marker neural/glial antigen 2 (NG2) in tumor sections. As NG2 is also expressed by tumor cells, CD31 and NG2 were co-stained to determine double-positive cells. Pericytes were then discriminated from NG2+ tumor cells by CD31 expression and morphology using the phenotyping tool of the Inform software (arrows, Figure 4C). Double positive cells were reduced in tumors of IL27Rα KO mice as was the ratio of CD31^+^NG2^+^ pericytes to CD31^+^ ECs (Figure 4D). These findings indicate reduced vessel maturation in tumors of IL27Rα KO compared to WT mice.

### Loss of IL-27 Signaling Enhances EC Sprouting, Proliferation, and Migration

**Figure 5:**
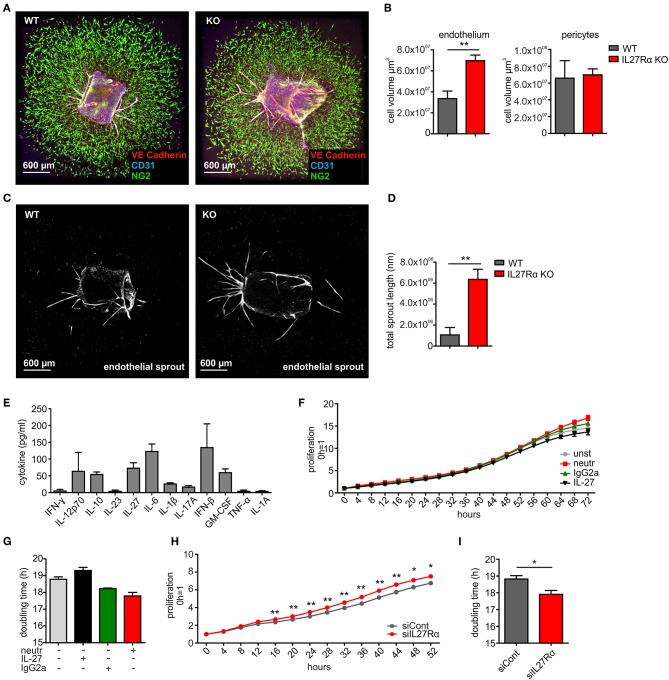
IL-27 restricts endothelial cell proliferation and sprouting. **(A–D)** Aortic ring assay was performed with aortas from wildtype (WT) and IL27Rα knockout (KO) mice, respectively. **(A)** Representative immunohistochemical stainings of sprouted aortas for endothelial cells (CD31, VE-Cadherin) and pericytes (NG2). **(B)** Sprout volume was analyzed using Imaris, considering volume of vascular and perivascular sprouting (*n* = 5). **(C,D)** Total endothelial sprout length analyzed with ImageJ is displayed (*n* = 5). **(E)** Cytokine production by bEnd5 endothelial cells was analyzed using Legendplex (*n* = 4). **(F–I)** bEnd5 endothelial cells were controls, treated with IL-27 neutralizing Ab, IgG2a, or IL-27 **(F,G)**, or transfected with siIL27Rα, or siControl (siCont) **(H,I)**. Proliferation was monitored for up to 72h. Time kinetics **(F,H)** and doubling time **(G,I)** are displayed (siCont *n* = 6, siIL27Rα *n* = 6, unst *n* = 3, IgG2a *n* = 4, IL-27 neutralizing Ab *n* = 4, IL-27 *n* = 4). Data are means ± SEM; *p*-values were calculated using one-sample *t*-test; *p < 0.05, **p < 0.01.

To analyze if a direct impact of IL-27 on vessels may explain the phenotype in IL27Rα KO mice, we first analyzed EC sprouting using aortic rings from IL27Rα WT and KO mice *ex vivo*. Sprouted aortic rings were stained for CD31, VE-cadherin and NG2. To analyze microvascular sprouting, Z-stacks were merged and the total volume of sprouted CD31 and VE-Cadherin expressing ECs and NG2 positive pericytes was determined (Figures 5A,B). Sprouting of IL27Rα KO ECs was significantly enhanced compared to WT ECs, whereas no differences in pericyte outgrowth occurred. Besides microvascular sprouting, the endothelial sprout length was significantly enhanced in aortic rings lacking IL27Rα (Figures 5C,D).

To explain alterations in EC sprouting, we next analyzed EC proliferation and migration. For this, the endothelial cell line bEnd5 was used. These cells constitutively produce IL-27 and are therefore suitable for IL-27 neutralization approaches (Figure 5E). Cells remained either untreated, were transfected with IL27Rα-specific siRNA compared to a non-targeting control (Supplementary Figure 5), received an IL-27 neutralizing antibody compared to an isotype control antibody, or were supplemented with IL-27 (Figures 5F–I). Proliferation was followed over a time course of up to 72 h and differences were analyzed at the endpoint. Untreated cells showed a proliferation slope of ~14.5 h and doubling time of 18.8 h. IL-27 treated cells showed a proliferation slope of 13.6 and a doubling time of 19.3 h (Figures 5F,G). This suggested mildly impaired proliferation upon IL-27 treatment. Interfering with IL-27 signaling by adding the IL-27 neutralizing antibody promoted proliferation compared to the IgG2a isotype control (Figures 5F,G). Differences in proliferation when IL-27 signaling was absent were stronger when siIL27Rα treated cells were used (Figures 5H,I). This may be due to the fact that the neutralizing antibody interferes with IL-27 p28, which by itself (then designated IL-30) can signal through the IL-6 receptor (35). Thus, the neutralizing antibody is less specific compared to IL27Rα-specific siRNA.

**Figure 6:**
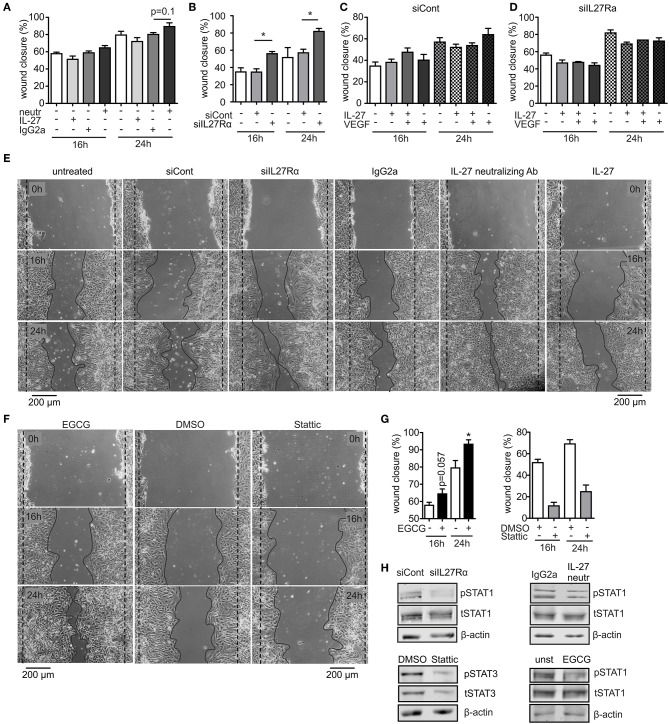
IL-27 signaling restricts EC migration. **(A–E)** bEnd5 endothelial cells were controls, transfected with siIL27Rα, siControl (siCont), or treated with a IL-27 neutralizing Ab (neutr), IgG2a, VEGF, or IL-27 and subjected to a wound healing assay. Quantification of wound closure after 16 and 24 h **(A–D)** and representative images **(E)** are shown (*n* = 4). **(F,G)** bEnd5 endothelial cells were controls, treated with DMSO, the STAT1 inhibitor Epigallocatechin gallat (EGCG), or the STAT3 inhibitor Stattic. Representative images **(F)** and quantification of wound closure after 16 and 24 h **(G)** are shown (*n* = 4). **(H)** bEnd5 endothelial cells were controls, transfected with siIL27Rα, siControl (siCont), or treated with a IL-27 neutralizing Ab, or IgG2a. Protein expression of phospho-STAT1 (pSTAT1) vs. total STAT1 (tSTAT1), and phospho-STAT3 (pSTAT3) vs. total STAT3 (tSTAT3) were determined after 24 h of the scratch assay using Western analysis (cropped blots, siCont *n* = 8, siIL27Rα *n* = 8, IgG2a *n* = 3, IL-27 neutralizing Ab *n* = 3; DMSO *n* = 4; Stattic *n* = 4; unst, *n* = 4; EGCG *n* = 4). Data are means ± SEM; *p*-values were calculated using one-sample *t*-test; *p < 0.05.

To analyze migration, we used a wound assay and monitored wound closure over time. Neutralizing IL-27 signaling significantly promoted wound closure compared to the IgG2a isotype control after 16 h, while the presence of IL-27 slowed wound closure. Wound areas treated with IL-27 neutralizing Ab were closed to roughly ~90% after 24 h, whereas wound closure in IgG2a isotype control samples reached only ~75% (Figures 6A,E). The difference in migration between IL-27 neutralizing Ab and IgG2a-treated samples was again stronger when an siRNA approach was used. A knockdown of IL27Rα significantly enhanced migration at 16 and 24 h compared to cells treated with a non-targeting control (Figures 6B,E). Additional stimulation with VEGF showed no further effect on wound closure (Figures 6C,D). IL-27 signals mainly via STAT1 and STAT3. To question whether these signaling pathways enhanced migration, the wound assay was performed in bEnd5 cells with STAT1 and STAT3 inhibitors. Stattic was used as a STAT3 inhibitor, while EGCG inhibits STAT1 (36). In the presence of EGCG wound closure was similarly enhanced compared to the situation seen in siIL27Rα treated cells (Figures 6F,G), while Stattic reduced EC migration compared to the control (Figures 6F,G). Importantly, STAT1 phosphorylation was reduced in cells treated with siIL27Rα, IL-27 neutralizing Ab and EGCG (Figure 6H). Our findings suggest STAT1 as a likely signaling pathway that attenuates EC migration downstream of the IL-27 receptor in ECs, and furthermore indicate that IL-27 may restrict functional angiogenesis by limiting EC migration, proliferation and sprouting.

## Discussion

In order to grow and survive, tumor cells show a high demand for nutrients and oxygen, and, thus, need to provoke angiogenesis. Tumor angiogenesis is considered to be fundamentally different from physiological angiogenesis. In tumors, excessive sprouting and vessel branching generates convolute and leaky vessels (28, 37). Anti-angiogenic therapy using vascular endothelial growth factor (VEGF)-targeting agents alone, or in combination with chemotherapy, normalized a disordered tumor vasculature rather than disrupting it (38). Rather, we suggest a third scenario in tumors, where compromised tumor vessels can be rendered even more dysfunctional, to again restrict tumor growth. In support of this hypothesis two recent studies showed that a loss of delta-like 4 (Dll4) increased in non-functional and convolute vessels, thereby reducing tumor growth (39, 40). We observed a similar phenomenon in our study when depleting IL27Rα in stromal cells. It would have been of interest to reduce dysfunctional angiogenesis to a certain degree to prove causality in our system, i.e., by neutralizing VEGF to normalize vessels. However, our data did not establish a functional interplay between IL-27 and VEGF signaling. Therefore, we refrained from testing this hypothesis. Importantly, our study in accordance with the studies of Noguera-Troise et al. and Ridgway et al. suggests that tipping the balance of angiogenesis in tumors toward both directions, vessel maturation or a loss of function might be suitable during tumor therapy.

A role of IL-27 in EC function and angiogenesis is currently underappreciated. Only one study demonstrated that IL-27 reduced tumor angiogenesis in a melanoma model and an *in vivo* angiogenesis assay. In this study, IL-27 elicited the production of CXCL9 and CXCL10 in human ECs (16). Although CXCL9 and CXCL10 are described as anti-angiogenic chemokines, their impact on the tumor vasculature under conditions of IL-27 treatment was not tested (16). We did not observe altered expression of CXCL9 or CXCL10 in endothelial cells isolated from WT or IL27Rα KO tumors (data not shown). We show that rather depletion of IL-27 signaling disturbs tumor angiogenesis. IL27Rα deficiency increased EC proliferation, migration and sprouting, which corroborates that IL-27 limits angiogenesis. It remains to be determined whether acute inhibition of IL-27 signaling would phenocopy the effects on the vasculature seen in IL27Rα KO mice. Functional vessels are needed for immune cells to infiltrate tumors (41, 42). If blood vessel are disturbed, immune cells, such as T cells, B cells, or NK cells are unable to enter the tumor. Dysfunctional vessels in IL27Rα KO mice may explain reduced immune cell numbers, particularly lymphocyte numbers in late stage tumors of KO mice, rather than reduced proliferation of these cells in tumors.

Blocking IL-27 signaling was without consequences regarding the number of activated or quiescence/resting ECs. However, activation of CD54^+^CD106^+^ and CD54^+^ ECs was increased in tumors of early stage KO mice. EC activation is a defined two-stage process. Type I EC activation occurs immediately after stimulation, when endothelial adhesion molecules, such as P-selectin emerge at the cell surface. Type II EC activation is a delayed process, whereupon E-selectin is induced at the cell surface and chemokines are released (43). If one of these two steps is uncontrolled, ECs can undergo morphological changes or become dysfunctional. In early stage tumors of KO mice, we detected increased markers for both activation states in CD54^+^CD106^+^ and CD54^+^ ECs. Significantly more P-selectin was expressed in CD54^+^CD106^+^ ECs in early stage tumors of KO mice. Also, E-selectin was over-abundant. Overexpression of both EC activation markers suggests unregulated EC activation, which might fit to enhanced migration and proliferation (43). Taken together vessels in tumors of KO mice are poorly perfused, malformed, and leaky, as observed upon FITC-BSA injection. Angiogenesis starts with detachment of pericytes from the vessels and terminates with pericyte recruitment for vessel stabilization and maturation (44, 45). Anti-pericyte treatments in tumor therapy causes vascular regression and inhibits tumor growth (46, 47). We detected less pericytes and an attenuated pericyte to ECs ratio in tumors of KO mice compared to WT mice. This points to vessel leakiness under these conditions.

EC proliferation and migration are important for angiogenesis (48). Within the first phase of angiogenic sprouting a few endothelial cells are selected, which lead the growing sprout (49). These ECs adapt a more invasive and migratory phenotype to migrate toward, e.g., VEGF gradients generated from tumor cells. Leading ECs are followed by a second subset of EC, which proliferate, elongate and form the lumen of new vessels. We show that the absence of IL-27 signaling enhanced endothelial and microvascular sprouting, whereas pericyte sprouting was unaffected. This suggests an altered ratio of sprouted pericytes relative to ECs, which fits to leaky vessels. When looking at mechanisms that may explain altered sprouting, we noticed enhanced migration and proliferation of ECs treated with a IL-27 neutralizing antibody or lacking IL27Rα. IL-27 signals via STAT1/3 and STAT signaling has been connected to angiogenesis previously. While STAT3 signaling was previously shown to promote angiogenesis, STAT1 is a negative regulator of angiogenesis (50, 51), which fits to our data. Inhibition of STAT3 using Stattic significantly lowered migration. Inhibition of STAT1 using EGCG (36) significantly increased EC migration, similar to the situation when blocking IL-27 signaling. Furthermore, neutralizing IL-27 reduced STAT1 signaling in ECs. These data suggest that IL-27 signaling restricts EC migration and thus, angiogenesis via STAT1. The downstream signals certainly need further investigation. Conclusively, our study reveals a so far unappreciated direct impact of IL-27 signaling on endothelial cells to alter angiogenesis.

## Data Availability Statement

All datasets generated for this study are included in the manuscript/Supplementary Files.

## Ethics Statement

The animal study was reviewed and approved by Hessian animal care and use committee.

## Author Contributions

AW, DS, and BB: conceptualization. AF, GC, RP, ES-F, and A-CF: methodology. AF, GC, RP, and AW: formal analysis. AF, GC, RP, ES-F, and A-CF: investigation. IF and BB: resources. AF, GC, RP, and AW: data curation. AF and AW: writing–original draft. AF, GC, RP, and AW: visualization. IF, DS, AW, and BB: supervision. IF, DS, and BB: funding acquisition. All authors: writing–review and editing.

### Conflict of Interest

The authors declare that the research was conducted in the absence of any commercial or financial relationships that could be construed as a potential conflict of interest.
