# Supplementary material for: IL27Rα Deficiency Alters Endothelial Cell Function and Subverts Tumor Angiogenesis in Mammary Carcinoma
Source: Front Oncol. 2019 Oct 4;9:1022. doi: 10.3389/fonc.2019.01022 (PMC6787910; doi:10.3389/fonc.2019.01022)
Supplement: Supplementary file 1 [file Data_Sheet_1.PDF]

Supplementary figures

Supplementary figure 1

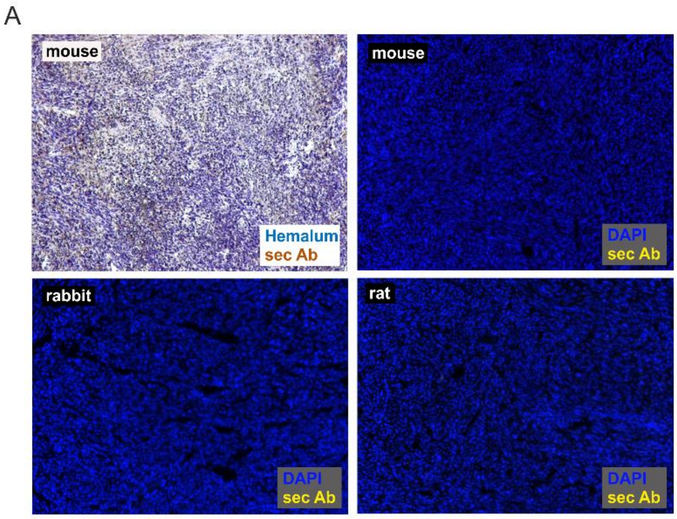

Supplementary figure 1

Supplementary figure 1 Secondary antibody controls. (A) Secondary antibody controls for mouse (HIF1α) were routinely tested with DAB staining. Secondary antibody controls for rat (αSMA), rabbit (Ki67 and CASP3) and mouse (CD31 and NG2) were likely routinely acquired together with DAPI.

Supplementary figure 2

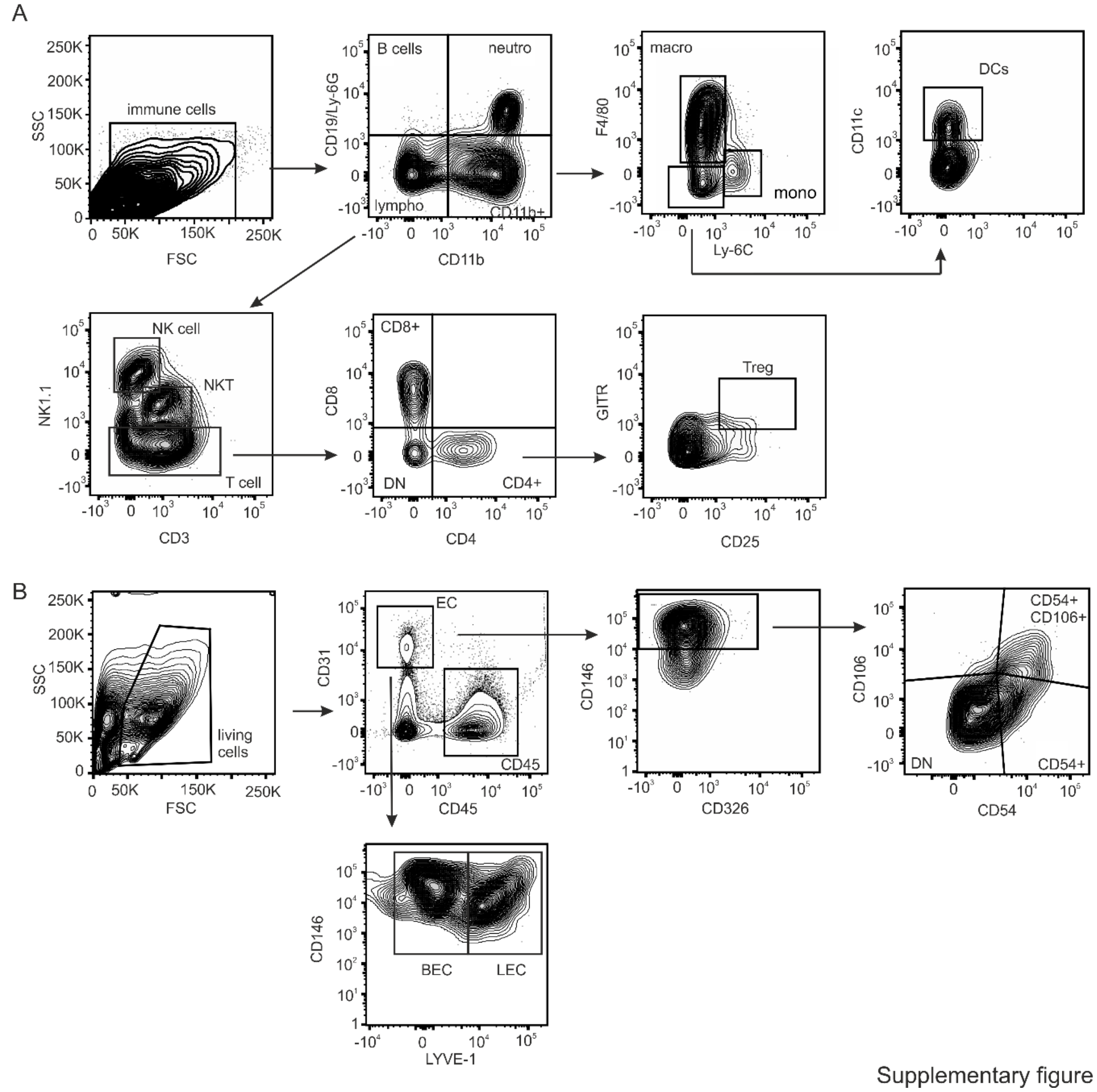

Supplementary figure 2

Supplementary figure 2 Gating strategy for immune and endothelial cells. (A) Gating strategy for flow cytometry. First, immune cells were selected. Within the immune cell population B cells, neutrophils (neutro), lymphocytes (lympho), and CD11b positive cells were distinguished. CD11b+ cells were further analyzed for macrophages (macro), monocytes (mono), and Ly-6C negative cells. Ly-6C- cells were gated against CD11c to determine dentritic cells (DCs). Lymphocytes were further analyzed for natural killer cells (NK cells), natural killer T cells (NKT cells), and T cells. Within the T cell population CD4 and CD8 positive cells were separated. CD4+ T cells were discriminated into regulatory T cells (Treg, GITR). (B) Gating strategy to discriminate

endothelial cells. First living cells were selected. Within this population, endothelial cells (EC) and immune cells (CD45) were discriminated. Within the EC population blood (BEC) and lymphatic endothelial (LEC) cells were analyzed. Within the BEC population (CD146) different surface marker were analyzed.

Supplementary figure 3

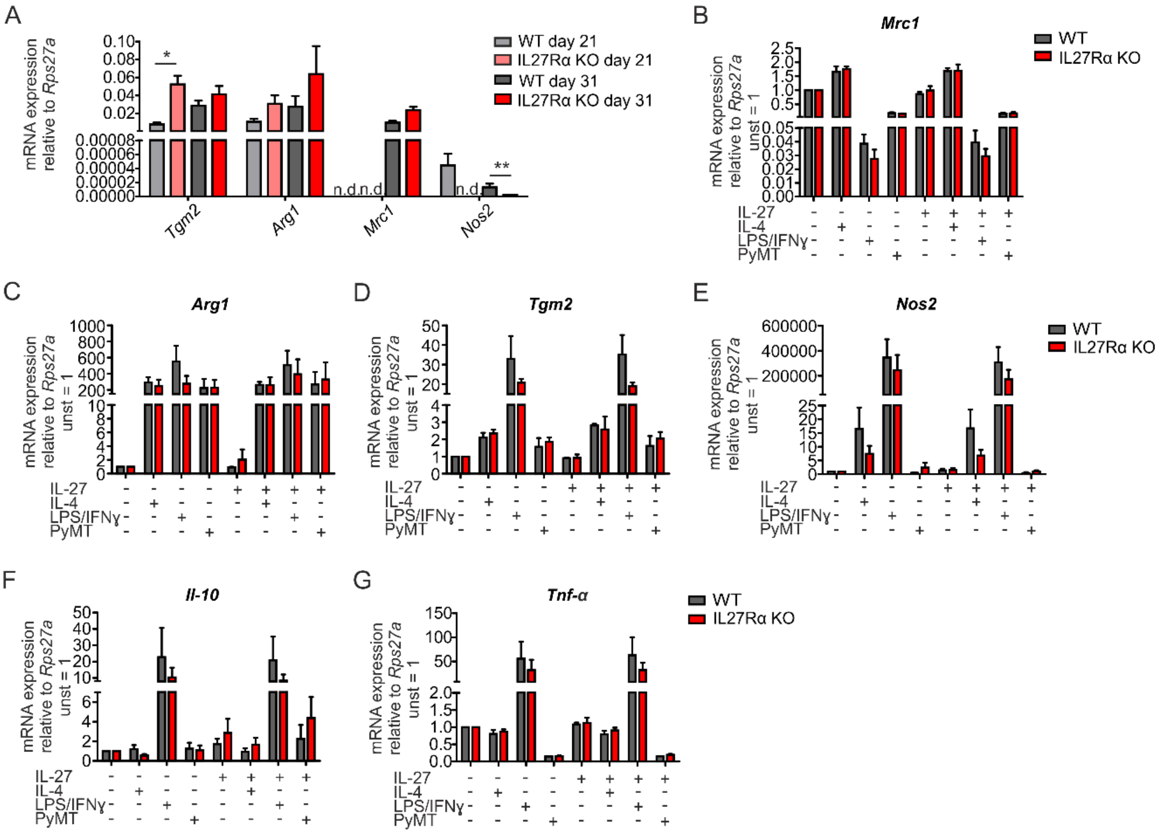

Supplementary figure 3

Supplementary figure 3 Macrophage polarization and IL-27 signaling. (A) Quantitative real time PCR from whole tumor mRNA was performed for *Tgm2*, *Arg1*, *Mrc1* and *Nos2* (WT day 21 n=4, KO day 21 n=4, WT day 31 n=6, KO day 31 n=5). (B-G) Bone marrow derived macrophages were generated and stimulated with IL-4 (20 ng/ml) for 24 h, LPS (100 ng/ml) + IFNɣ (10 ng/ml), IL-27 (100 ng/ml) or were cocultured with PyMT cells for 48 h. Expression of macrophage polarization markers *Mrc1* (B), *Arg1* (C), *Tgm2* (D), and *Nos2* (E), as well as cytokines *Il-10* (F), *Tnf-α* (G) at mRNA level were analyzed (WT n=4, KO n=4). Data are means ± SEM, p-values were calculated using one-sample t test; *, $p < 0.05$, **, $p < 0.01$; n.d. not detected.

Supplementary figure 4

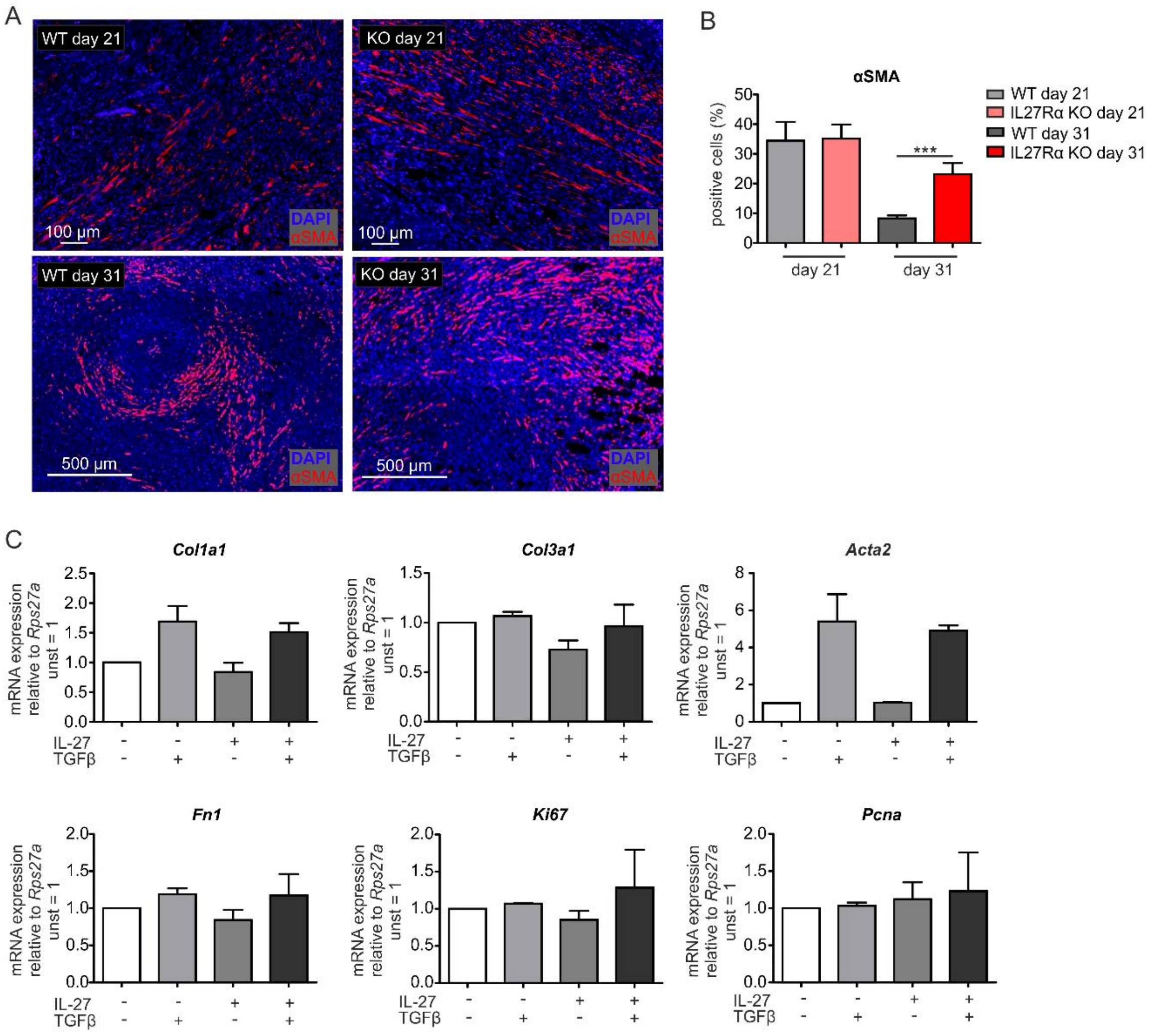

Supplementary figure 4

Supplementary figure 4 IL-27 does not directly affect fibroblast activation or proliferation. (A) PyMT breast cancer cells were transplanted into mammary glands of IL27Rα wildtype (WT) and knockout (KO) mice. Tumors were harvested after 21 or 31 days. Representative stainings of fibroblasts (WT n=8, KO n=10). (B) Quantification of data shown in A using inForm Software. (C) Quantitative real time PCR from 3T3 fibroblasts stimulated with IL-27 (100 ng/ml) and/or TGFβ (10 ng/ml) for 72 h for *Col1a1, Col3a1, Acta2, Fn1, Ki67,* and *Pcna* (n=2)*.* Data are means ± SEM; p-values were calculated using one-sample t test; ***, $p < 0.001$.

Supplementary figure 5

A

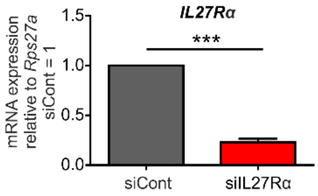

Supplementary figure 5

Supplementary figure 5 IL27Rα mRNA expression after the depletion of IL27Rα. (A) Quantitative real time PCR analysis *IL27Rα* and *Rps27a* from siRNA treated bEND5 cells (n=4). Data are means ± SEM; p-values were calculated using one-sample t test; ***, $p < 0.001$.
